# Internet self-efficacy moderates the association of information technology ability with successful ageing among older employees in three African samples

**DOI:** 10.1007/s10433-024-00827-9

**Published:** 2024-10-18

**Authors:** Nestor Asiamah, Sylvester Hatsu, Frank Frimpong Opuni, Faith Muhonja, Confidence Chinwe Opara, Sarra Sghaier, Emelia Danquah

**Affiliations:** 1 Division of Interdisciplinary Research and Practice, School of Health and Social Care, University of Essex, Colchester, Essex CO4 3SQ UK; 2 Africa Center for Epidemiology, Department of Geriatrics and Gerontology, P. O. Box AN 18462, Accra, Ghana; 3 Department of Computer Science, Accra Technical University, BarnesRoad, P. O Box GP 561, Accra Metro, Accra, Ghana; 4 Department of Marketing, School of Business, Accra Technical University, Barnes Road, P. O Box GP 561, Accra Metro, Accra, Ghana; 5 Department of Community Health, School of Public Health, Amref International University, P. O. Box 27691 – 00506, Nairobi, Kenya; 6 Department of Banking and Finance, College of Management Sciences, Michael Okpara University of Agriculture Umudike, Umuahia, Abia State Nigeria; 7 Research Directorate, Koforidua Technical University, Eastern Region, Post Office Box KF-981, Koforidua, Ghana

**Keywords:** Information technology, Successful ageing, Self-efficacy, Older adults, STROBE, Moderation, Africa

## Abstract

The literature suggests that two distinct competencies [i.e., information technology ability (ITA), and internet self-efficacy] are necessary for the effective use of information technologies for successful ageing, but no study has examined the association of these skills on successful ageing and its domains (i.e., illness avoidance, functioning, and engagement with life). This study investigated whether Internet Self-Efficacy (ISE) moderates the potential association of ITA with successful ageing. The study adopted a cross-sectional design based on the Strengthening the Reporting of Observational Studies in Epidemiology (STROBE) checklist and includes measures against confounding and common methods bias. The participants were 1186 older workers aged 50 years or older in three African countries (Kenya = 350; Nigeria = 260; Ghana = 576). The minimum sample size necessary was calculated, and data were analysed with hierarchical linear regression analysis. After controlling for the covariates, ITA and ISE had a positive association with successful ageing as well as illness avoidance, functioning, and engagement with life in the whole sample. ITA was also positively associated with successful ageing and its domains in Kenya and Ghana. The associations of ITA with successful ageing and its domains were positively moderated by ISE in the whole sample and within each of the three samples. Higher ITA can be associated with successful ageing among working older adults, and ITA positively influences successful ageing at different levels of ISE. Older adults with high ITA are more likely to avoid illness at a higher ISE.

**Supplementary Information:**

The online version contains supplementary material available at 10.1007/s10433-024-00827-9.

## Introduction

As population ageing intensifies, the burden of disease and its associated healthcare expenditure are expected to increase globally. If individuals are not supported to age in good health, population ageing may become a national burden accompanying a high old-age dependency. Information technologies play a key role in successful ageing (Fang et al. 2019; Pruchno 2019; Sghaier et al. 2022) though their use can accompany risks such as stress, cyberbullying, and account hacking if abused or used to engage with fraudulent people (Carter 2013; Lim and Choi 2017; van Zoonen and Rice 2017). Thus, the contribution of information technologies to successful ageing would depend on whether older adults can effectively and safely use information technologies. We refer to this ability to use information technologies as Information Technology Ability (ITA).

Another skill upheld as a basic determinant of information technology use among older adults is self-efficacy, which is encapsulated in the term “internet self-efficacy” (Jokisch et al. 2020; Sun 2008). ITA and Internet Self-Efficacy (ISE) are both competencies but are different because the former is a measure of the capability to use information technologies in general (Yu and Chao 2014), whereas the latter is a belief in one’s ability to use the internet (Jokisch et al. 2020). Information technologies used by older adults (e.g., WhatsApp, social media, and Google navigation application) depend on the internet (Liu et al. 2016; Yusif et al. 2016), so the ability to use these technologies requires knowledge about how to activate and access internet services. Suffice it to say that older adults’ ISE (which concerns their use of the internet) can facilitate ITA. If so, ISE may interact with ITA to influence successful ageing. This moderating role can be more significant among working older adults who have access to information technologies at work and learn how to use these technologies through workplace human development programmes (e.g., training). This role has not been evaluated though it has important implications for workplace ageing policy. Because the global workforce is ageing quickly, an elucidation of these implications is imperative.

Non-availability of empirical evidence on the above moderating role is a major gap in the literature for some reasons. Firstly, the “digital divide” (Fang et al. 2019; Pruchno 2019), which is a maxim emphasizing relative underutilisation of information technologies in older populations, can be bridged with empirical evidence regarding the individual and joint roles of ISE and ITA in successful ageing. Secondly, studies in gerontology have not sufficiently emphasised the role of information technology skills in successful or healthy ageing (Pruchno 2019; Sghaier et al. 2022), which is problematic because the ability to use technologies is a prerequisite to technology-enabled ageing (Sghaier et al. 2022; Yu and Chao 2014), and there have been calls for interventions equipping older adults with these skills (Pruchno 2019; Sghaier et al. 2022). Finally, a study in Germany (Jokisch et al. 2020) suggests that ISE can be associated with the avoidance of information technology among older adults. For instance, ISE may be associated with high formal education and awareness about the risks of information technologies. Older adults with higher ISE who are aware of these risks would avoid information technologies. Thus, ISE may not encourage information technology use towards successful ageing in every context. The above study also suggests that ISE can be more strongly associated with information technology avoidance among older adults with higher ITA. The foregoing counterintuitive evidence suggests that ISE and ITA may not jointly support successful ageing, and thus, warrants an assessment of the above moderating role for the first time to consolidate the available evidence and lessons for practice.

This study, therefore, assessed the above potential moderation with two research questions: (1) is ITA associated with successful ageing and its domains (i.e., illness avoidance, functioning, and engagement with life), and (2) does ISE moderate the association of ITA on successful ageing? Our aim was not to compare these associations between countries but to assess them for the whole sample and for individual countries. This approach allowed us to disaggregate the evidence to provide insight into each country.

A systematic review (Aggarwal et al. 2020) has revealed a paucity of studies focused on the role of information technologies in ageing in Africa. This situation is problematic since the population in Africa will age rapidly and African stakeholders need to prepare to support their older adults to use information technologies. With this study, we hope to create an impetus for accelerated research within the gerontology-technology subfield in Africa and contribute towards bridging the research gap between Africa and the rest of the world within this field.

## Literature review

### Overview of the key concepts

Information technologies (e.g., internet-driven applications such as WhatsApp, Twitter, and emails) have become important facilitators of daily living. An increasing array of these technologies is used to perform social and physical functions such as walking and communication (Kim et al. 2017; Teixeira-Santos et al. 2022; Yusif et al. 2016). Many individuals with physical and cognitive limitations live a normal life by using information technologies to access services (e.g., healthcare) (Nam et al. 2019; Schlomann et al. 2020). In older populations, quality of life can be an outcome of how well information technologies are used to engage with social networks and support mobility (Nam et al. 2019; Sghaier et al. 2022), suggesting that many older adults cannot live a normal life without information technologies. Yet, having relevant information technologies does not necessarily provide benefits for successful ageing; older adults need ITA to be able to effectively and safely use information technologies to achieve pro-health goals.

ITA is the ability to use information technologies or try out new versions of these technologies to perform a task (Sghaier et al. 2022; Yu and Chao 2014). This definition was an outcome of a psychometric test (Yu and Chao 2014) in which ITA was proxied with three constructs, namely internet use assessment, packaged software use assessment, and personal innovativeness attitude. This psychometric test has produced one of several tools available for measuring ITA. An example is the Computer Proficiency Question (Boot et al. 2015), which was developed to measure a wide range of competencies among older adults. Another tool is the Subjective Technology Adaptivity Inventory (Kamin and Lang 2013) constructed to assess perceived personal adaptivity in technological environments. The availability of these tools signifies the importance of older people’s information technology proficiency.

We chose the psychometric tool of Yu and Chao (2014) to measure ITA in this study since it is well suited for the Ghanaian context. According to this tool, internet use assessment is the ability to use the internet to obtain, retrieve or send information (Yu and Chao 2014). This encompasses the ability to send and read emails with or without attached files. Packaged software use assessment is the ability to use packaged software (e.g., Microsoft Office, WhatsApp, and Twitter) and describe functions performed by these applications (Sghaier et al. 2022). This includes being able to edit documents with relevant software. Personal innovativeness attitude is the willingness of a person to try out new technologies (Sghaier et al. 2022; Yu and Chao 2014), which is necessary for developing expertise in using technologies to achieve goals towards successful ageing. Even so, the influence of ITA on successful ageing can be complemented by ISE.

Many definitions of ISE draw on seminal works (Bandura 2004; Marcus et al. 1998). *People's belief in their ability to use the internet to perform a task* (Torkzadeh and Van Dyke, 2002) is one of these definitions. ISE has also been defined as people’s confidence in using the internet to perform social functions (Tsai et al. 2011) and people’s perception of their ability to use the internet (Chuang et al. 2015). We recognise the differences between these and other definitions; hence, we adopt the definition of Torkzadeh and Van Dyke for its consistency with the scale used to measure ISE in this study. Most studies recognise ISE as a measure of people’s belief in their ability to use the internet (Jokisch et al. 2020, 2022) in agreement with the definition of Torkzadeh and Van Dyke. Bandura and Marcus et al. also recognise ISE as a belief in one’s potential to perform a task with technologies and argue that ISE can be a facilitator of ITA. Notably, ISE can complement ITA to engender successful ageing.

Successful ageing is the avoidance of illness and the maintenance of functioning as well as engagement with life in old age (Bowling and Dieppe 2005). It is alternatively defined as the maintenance of high independence in the community by living at an advanced age, continuing to function well at home and remaining mentally alert (Peel et al. 2004). Though these definitions came from different contexts and therefore slightly differ, there is a consensus among researchers that successful ageing is about avoiding illnesses, maintaining functioning, and remaining engaged with life (Peel et al. 2004; Tong et al. 2022; Zacher 2015). This framing of successful ageing is consistent with Rowe and Khan’s framework (Rowe and Kahn 1997) and a previous psychometric test in which successful ageing was measured as a construct of four domains, namely illness avoidance, functioning, caring engagement (i.e., caring for others), and productive engagement (i.e., one’s contribution to society in general), with the last two domains measuring engagement with life (Ng et al. 2011; Tong et al. 2022). Thus, successful ageing is characterized by the maintenance of optimal health, functional ability, and engagement with life.

We propose that, based on the foregoing definitions, successful ageing is an indicator of individual performance that could translate into organizational productivity. Productivity at the workplace and in other spheres of life depends on whether the individual avoids illnesses and maintains functioning and engagement with life. If, for instance, employees fail to avoid illnesses, they are unlikely to achieve optimum productivity at work; they may have a lot of absences due to ill health and fail to achieve work targets, which would translate into low individual and organizational productivity. Successful ageing reduces the probability of employees retiring too early and earning gratuity due to ill health. The failure of ageing employees to avoid illness could result in accumulated expenditures from organizational health insurance payments. Our recognition of successful ageing as a performance indicator is consistent with studies (Collins et al. 2005; Mills et al. 2007; Terry 2022) that acknowledge workplace health promotion as a productivity improvement agenda. It is worth noting that recognising successful ageing as a core performance indicator is warranted by the ageing of the world’s population. This recognition would enable organizations and employers to design workplace ageing policies, including those aimed at the improvement of ITA and ISE.

### Theoretical framework and model

We operationalise successful ageing as a worker performance indicator based on the Job Characteristics Theory (JCT), which posits that the individual needs a variety of abilities to achieve individual performance outcomes such as job performance, motivation, and satisfaction (Asiamah 2017; Ma et al. 2022). These outcomes can be positively associated with health in old age (Dagenais-Desmarais et al. 2018; Faragher et al. 2013; Stronks et al. 1997), which means their antecedents such as ITA can positively influence successful ageing. Employees are required to use information technologies to perform job tasks such as sending emails and using the computer to prepare files. The use of information technologies outside the workplace to access services (e.g., healthcare) and engage with life can directly contribute to successful ageing or have a spill-over effect on the above performance indicators. In today’s digital age, these performance indicators can be expected to be significantly influenced by information technologies. This influence, by the argument of JCT, is contingent on ITA. Thus, ITA can influence successful ageing directly (e.g., by supporting functioning, engagement with life, and access to healthcare) or indirectly through the above performance outcomes. We would want to use the Lifespan Theory of Control (LTC) to explain why ITA can positively influence successful ageing.

**Fig. 1:**
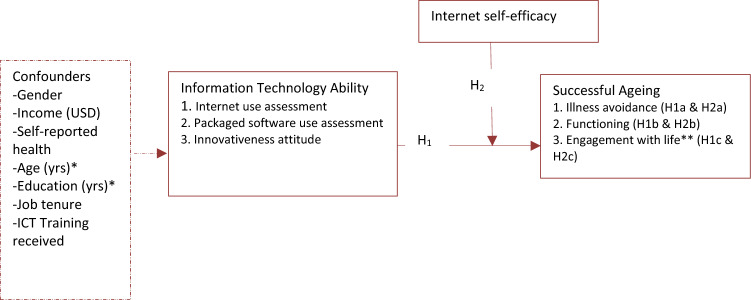
A model of modifiability of the association of information technology ability with successful ageing by internet self-efficacy. *Ultimate confounders incorporated into the ultimate models; **the domains “caring engagement” and “productive engagement” were combined into “engagement with life” based on an exploratory factor analysis; broken arrow indicates confounding; USD—United States Dollars; ICT—information communication technology; H1—Information technology ability is associated with successful ageing; H1a Information technology ability is associated with illness avoidance; H1b—Information technology ability is associated with functioning; H1c—Information technology is associated with engagement with life; H2—internet self-efficacy moderates the association of information technology ability with successful ageing; H2a—internet self-efficacy moderates the association of information technology ability with illness avoidance; H2b—internet self-efficacy moderates the association of information technology ability with functioning; H2c—internet self-efficacy moderates the association of information technology ability with engagement with life.

The LTC proposes that behaviours performed over the life course are driven by the desire to exert control over one’s environment. The two primary concepts of the theory are *primary control* and *secondary control* (Heckhausen and Schulz 1995), with the former referring to behaviours aimed at the external environment and involving efforts to influence the world in harmony with the individual’s current needs and desires. Primary control can be selective or compensatory. In selective primary control, individuals invest behavioural resources such as effort and skills in their pursuit of a goal. To illustrate, an individual may apply ITA to use information technologies to perform pro-health goals, including healthcare utilisation and exercise adherence. This thinking is supported by several studies (Aggarwal et al. 2020; Nakagomi et al. 2022; Szabo et al. 2019) that have confirmed a positive association of information technology use with health indicators (e.g., quality of life and self-reported health) among older adults. Since all the domains of successful ageing are health indicators, we envisage that higher ITA, which is necessary for technology use, would be associated with higher successful ageing (hypothesis 1; see Fig. 1).

*Compensatory primary control* is required when internal behavioural resources are insufficient for achieving a goal, which necessitates the utilisation of external resources to optimise one’s ability to perform a behaviour (Heckhausen and Schulz 1995; Schulz and Heckhausen 1996). For instance, an individual may need guidance or training (i.e., an external resource) to improve ITA for using information technologies. Research has shown that training as an external resource can enhance ISE (Lagana 2008) and can more easily be accessed at work. As a personal resource co-exiting with ITA (as asserted by the JCT), ISE can facilitate the utilisation of ITA and, thus, predict individual performance including successful ageing. We contend that people without sufficient ISE are unlikely to put their ITA to use, which explains why ISE can complement ITA to support successful ageing. As shown in Fig. 1, therefore, ISE can positively moderate ITA to influence successful ageing (hypothesis 2), especially in an organizational context where training as an external resource can be more easily accessed and maintained.

ITA can also influence ISE positively, which means that the link between ITA and ISE can be bi-directional. It is possible for older adults’ belief in their ability to use information technologies (i.e., their ISE) to increase as their use of these technologies increase. Yet, this belief is necessary for using information technologies for the first time and in the early period of information technology adoption. This argument is corroborated by studies (Ariff et al. 2012; Jokisch et al. 2022) that have shown that ISE is a determinant of information technology adoption. Thus, at the early stages of information technology adoption, the effect of ISE on ITA is more probable compared to the effect of ITA on ISE. Older adults in Africa are in their early years of information technology adoption since they got access to information technologies relatively recently (Ejiaku 2014; Udo and Edoho 2000). In an African context, therefore, an assessment of the foregoing moderating role implicit in the effect of ISE on ITA is imperative.

The above moderation may not be consistent across contexts and time partly because of the LTC’s perspective concerning *secondary control*, which assumes that people can lose primary control or the ability to achieve goals (e.g., using technologies for health) in later life. This viewpoint implies that people may lose ISE and ITA as they age, making it less possible for these two variables to interact on successful ageing. People may also encounter unexpected events (e.g., become physically disabled) or lose internal resources (e.g., physical functional ability) for pursuing goals as they age. Whether individuals can maintain behaviours depends on their ability to overcome these unexpected events by trading off less relevant goals for more important ones (Schulz and Heckhausen 1996). This ability, for instance, sustains the utilisation of external resources (e.g., training and technologies) to maintain health. Hence, people with high ISE and ITA but without the ability to retain primary control may be unable to effectively use information technologies for successful ageing. This reasoning implies that ISE and ITA may not positively influence successful ageing in older age.

The moderating role shown in Fig. 1 means that ISE can strengthen the positive association of ITA with successful ageing. Testing this relationship in this study would provide lessons for experimental studies, which may evidence a need for organizations to adopt human development programmes to support employees to maintain primary control over technology use in response to potential losses of personal resources in ageing. The presence of the potential confounders in Fig. 1 is our recognition of some other variables influencing the link between ITA and successful ageing. These confounding influences have been confirmed or reported by empirical studies and systematic reviews (Liao et al. 2017; Peacock and Künemund 2007; Slegers et al. 2012), which necessitated their consideration in this study.

## Methods

### Design

**Fig. 2:**
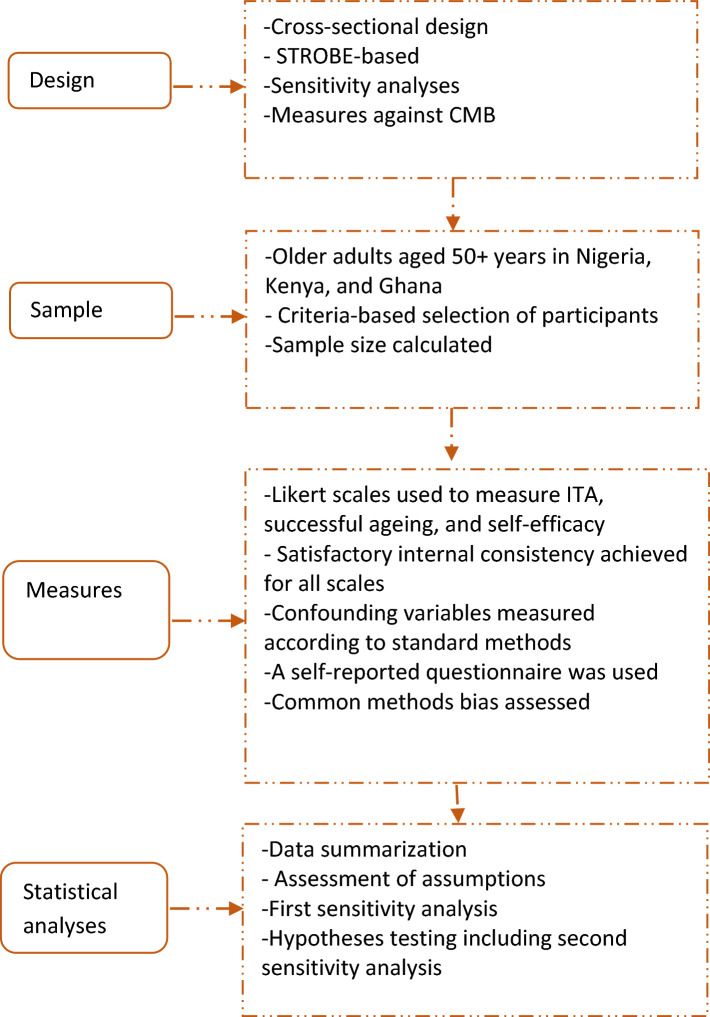
A flowchart of the STROBE-based design. *Note* *CMB* common methods bias, *ITA* information technology ability

This study employed a cross-sectional design based on the STROBE (i.e., Strengthening the Reporting of Observational Studies in Epidemiology) checklist. As Fig. 2 illustrates, it involved steps against common methods bias and the use of a hierarchical linear regressing (HLR) approach.

### Participants and selection

The participants were older adults aged 50 years or older who were working in both the manufacturing and service sectors in three countries (i.e., Nigeria, Kenya, and Ghana). The participants were selected based on three criteria: (1) being an employee in any organization; (2) having a minimum of basic education (i.e., basic school leaving certificate) as an indicator of the ability to speak in English, the medium in which the survey was administered, and (3) availability and willingness to voluntarily participate in the study. Employees in Ghana, Kenya, and Nigeria, regardless of their job ranks or category, have at least a basic educational qualification, except in the informal sector where people can work without any educational qualification. As such, only employees from the informal sector were excluded by the second criterion above.

Four researchers (i.e., SH, FFO, FM, and COC) and their research assistants selected the participants based on the above criteria in Accra (Ghana), Nairobi (Kenya), and Umuahia (Nigeria) through structured interviews. No sampling frame was available; hence, individuals were contacted and screened in their organizations or at community centres including supermarkets and malls. We utilised the G*Power software and recommended statistics (power = 0.8; *α* = 0.05; effect size = 0.2) from a related study (Sghaier et al. 2022) to calculate the minimum sample size necessary. The minimum sample reached for HLR with a maximum of 11 predictors was 95. To maximise the power of our tests, nevertheless, we collected data from all individuals (*n* = 1209; Nigeria = 271; Kenya = 363; Ghana = 575) who met the inclusion criteria.

### Measures

#### Successful ageing

The dependent variable, successful ageing, was measured with an 18-item scale adopted in whole with five descriptive anchors (i.e., 1—strongly disagree, 2—disagree, 3—somewhat agree, 4—agree, and 5—strongly agree) from a previous study (Ng et al. 2011). This scale was developed to measure successful ageing according to the definition of Rowe and Kahn (Rowe and Kahn 1997). This scale was associated with three domains [i.e., illness avoidance (4 items), functioning (5 items), and engagement with life (9 items)] with satisfactory Cronbach’s *α* values ≥ 0.7 (whole scale = 0.87; Nigeria = 0.88; Kenya = 0.85, and Ghana = 0.85). Online appendix A1 shows items of the successful ageing scale used.

#### Information technology ability

The independent variable, ITA, was measured with a standard 13-item scale adopted in whole with its five descriptive anchors (i.e., 1—strongly disagree, 2—disagree, 3—somewhat agree, 4—agree, and 5—strongly agree) from a previous study (Yu and Chao 2014). This accompanies three domains discussed earlier in this study [i.e., internet use assessment (5 items), packaged software use assessment (4 items), and personal innovativeness attitude (4 items)] and produced satisfactory Cronbach’s *α* values ≥ 0.7 (whole scale = 0.89; Nigeria = 0.88; Kenya = 0.94; Ghana = 0.80) in the current study and was, therefore, internally consistent. Online appendix A2 shows the scale for measuring ITA.

#### Internet self-efficacy

ISE was measured with an 8-item standard scale with six descriptive anchors (i.e., 1—strongly disagree; 2—moderately disagree; 3—slightly disagree; 4—slightly agree, 5—moderately agree, and 6—strongly agree) adopted in whole from a previous study (Sun 2008). It produced a satisfactory internal consistency (i.e., Cronbach’s *α* = 0.90; Nigeria = 0.88; Kenya = 0.93; Ghana = 0.82). Online appendix A3 shows the scale for measuring ISE.

#### Scoring of the scales

Higher scores on the above scales represent higher levels of the respective variables they measure. The maximum scores of successful ageing, ITA, and ISE are 90, 65, and 48, respectively. Based on previous research (Asiamah et al. 2022; Sghaier et al. 2022), data on the scales were generated by summing up scores from their corresponding items.

#### Potential confounders

Gender (male—1; female—2), whether ICT training was received (no—1; yes—2), and self-reported health (poor—1; good—2) were measured as categorical dichotomous variables. Recognising that older adults may have difficulties recalling past events, ICT training was measured as a dichotomous variable by asking the participants to report whether they had received any on-the-job training aimed at equipping them with ICT skills in the past year. Categorical variables were coded into dummy-type variables, with one group set as a reference. Income was measured as the individual’s gross monthly pay in United States dollars. Education was measured as years of schooling, whereas job tenure was measured as the number of years participants had worked in their current organization. Like income, education, and job tenure, age was measured as a discrete variable. This variable was then coded into a dummy-type variable to support regression analysis.

#### Instrumentation and common methods bias

A self-reported questionnaire was utilised to collect data. The questionnaire had a preamble of ethical considerations, the research aim, and the instructions for completing the survey. Scales and measures were presented in two blocks; the first block presented scales for measuring ITA, successful ageing, and ISE, whereas the other block captured questions measuring the potential confounding variables and ICT training. To avoid common methods bias, we followed recommended procedural and statistical approaches (Podsakoff et al. 2003). As part of the procedural approach, we presented each scale as a distinct section of the questionnaire by presenting its unique descriptive anchor, context, and instructions for survey completion. Secondly, we avoided using bipolar scales (e.g., − 3 to + 3) and used verbal labels instead of numeric labels or anchors as recommended (Podsakoff et al. 2003). Herman’s one-factor method was utilised to assess common methods bias through an exploratory factor analysis based on maximum likelihood (Kock et al. 2021; Sghaier et al. 2022). This statistical technique produced a satisfactory factor structure with at least two factors for the three scales as follows: successful ageing [3 factors; factor loadings ≥ 0.5], ITA [3 factors; factor loadings ≥ 0.5], and ISE [2 factors; factor loadings ≥ 0.5]. Moreover, the first factor for each scale produced a variance of less than 40% as recommended (Kock et al. 2021). Thus, common methods bias was absent in the data.

#### Ethics and data collection

This study received an institutional ethics clearance (003-11-2022-ACE) after the study’s protocol was reviewed by the ethics committee of the Africa Centre for Epidemiology. All ethical guidelines and procedures were followed in the interest of the human participants, including researchers. All the participants provided written informed consent before participating in the study. The four researchers serving as country coordinators and their research assistants hand-delivered self-administered questionnaires at the locations where the participants were recruited. Some participants completed and handed back the questionnaire instantly, whereas others were allowed to return completed questionnaires after two weeks. Many of the participants did not return their surveys after two weeks, so they were given two to five extra weeks to return their completed questionnaires. Data were collected over seven weeks (3rd November—23rd December 2022). Out of the 1209 questionnaires returned, 1186 were analysed; 23 were discarded because they were either not completed at all or were completed halfway.

## Analytical strategy

The data were analysed with SPSS 28 (IBM SPSS Inc., New York, USA) in three phases. Phase 1 was an exploratory analysis in which the data were summarised, and relevant assumptions governing the use of HLR analysis were assessed. Descriptive statistics were used to summarise the data and to identify missing items. The missing data were less than 4%, were associated with only two of the potential confounding variables (i.e., age and income), and were randomly distributed; hence, they were not removed (Asiamah et al. 2022). Five assumptions (e.g., linearity of the associations and normality distribution of the data) for HLR were subsequently assessed following recommended methods previously employed (Bempong and Asiamah 2022; Rezai et al. 2009; Sghaier et al. 2022). Online appendix B shows the five assumptions and steps taken to assess and meet them.

Phase 2 of the analysis aimed to compute interaction terms and define rules of thumb for assessing the moderating role. We utilised a method recently applied (Montoya 2019; Van Fossen et al. 2022) at this phase to assess moderation. As part of this technique, an interaction term (i.e., ITAxISE) was first computed using the compute function following previous studies (Montoya 2019). ITAxISE represents the interaction between ISE and ITA, and ISE was centred before this term was computed. As part of our moderation assessment, we examined the association between this interaction term and ITA as well as its domains.

Phase 3 was aimed at testing the primary hypotheses, including the moderating role of interest. Firstly, Pearson’s bivariate correlations were computed and assessed with the consolidated data as a basis of the HLR analyses. The non-adjusted models were tested by assessing the association of ITA and ISE with successful ageing and its three domains without incorporating the confounders. Subsequently, the adjusted (ultimate) models were fitted by incorporating the confounders and interaction term (i.e., ITAxISE) in the non-adjusted models. Models were fitted on the consolidated data and on specific countries. Following previous research (Asiamah et al. 2022; Bempong and Asiamah 2022), we performed a sensitivity analysis by comparing the standardized regression weights between the non-adjusted and adjusted models. The statistical significance of the results was detected at a minimum of *p* < 0.05.

## Findings

Table 1 shows summary statistics on the study variables. About 52% (*n* = 616) of the participants were men, and 78% (*n* = 930) of them were full-time workers. The average age of the participants was about 56 years (Mean = 56.43; standard deviation = 6.35). Table 2 shows the correlation coefficients among the relevant variables. ITA was positively correlated with successful ageing (*r* = 0.581; *p* < 0.001; two-tailed) and its domains, namely illness avoidance (*r* = 0.471; *p* < 0.001; two-tailed), functioning (*r* = 0.486; *p* < 0.001; two-tailed), and engagement with life (*r* = 0.51; *p* < 0.001; two-tailed). ISE was strongly correlated with ITA (*r* = 0.768; *p* < 0.001; two-tailed) and successful ageing (*r* = 0.595; *p* < 0.001; two-tailed), which reflected a possibility of ISE interacting with ITA on successful ageing.

**Table 1:** Summary statistics on variables included in the analyses (*n* = 1186)

Variables	Group	n/Mea*n*	%/SD
*Categorical variables*			
Country	Kenya	350	29.51
	Nigeria	260	21.92
	Ghana	576	48.57
Gender	Male	616	51.94
	Female	570	48.06
ICT training received (on the job)*	Training not received	632	53.29
	Training received	554	46.71
Self-reported health	Poor	192	16.19
	Good	994	83.81
	Total	1186	100.00
*Continuous variables*			
Information Technology Ability	–	45.68	10.14
Illness avoidance	–	13.72	3.62
Functioning	–	17.89	3.87
Engagement with life	–	32.96	6.75
Successful Ageing	–	64.56	12.11
Internet self-efficacy	–	32.62	8.82
Job tenure (yrs)	–	12.02	7.86
Income (USD)	–	216.02	190.41
Education (yrs)	–	16.50	4.18
Age (yrs)	–	56.43	6.35

*USD* US dollars, *ICT* information communication technology, *SD* standard deviation, *n* frequency, *ICT training was a measure of whether older adults had received at least one ICT training on the job over the past year, –Not applicable, the mean and SD apply to continuous variables only, whereas frequency and % apply to categorical variables only

**Table 2:** Bivariate correlations between information technology ability, successful ageing, internet self-efficacy, and the confounders (*n* = 1186)

Variable	1	2	3	4	5	6	7	8	9	10	11	12	13
1. ITA	1	.768**	.581**	.471**	.486**	.510**	−.068*	−.118**	0.01	−0.039	.116**	−.089**	.242**
2. ISE		1	.595**	.529**	.444**	.529**	−.059*	−.139**	−.075*	−.061*	.062*	−.113**	.220**
3. Successful Ageing			1	.740**	.857**	.905**	0.037	−.096**	−.086**	−0.039	−0.026	−.110**	.250**
4. Illness avoidance				1	.576**	.461**	0.036	−.102**	−.084**	−.060*	−.060*	−.118**	.155**
5. Functioning					1	.655**	0.046	−.084**	−0.056	−0.021	0.003	−.064*	.223**
6. Engagement with life						1	0.02	−.070*	−.077**	−0.026	−0.016	−.097**	.238**
7. Gender (ref—men)							1	−.114**	−0.028	−.077**	−0.043	−0.044	−0.017
8. Job tenure (yrs)								1	.132**	.384**	.275**	.130**	−0.019
9. ICT Training received (ref—no)									1	.173**	0.04	−0.028	0.008
10. Income (USD)										1	.289**	.141**	−0.028
11. Education (yrs)											1	.139**	−0.011
12. Age (yrs)												1	−.059*
13. SRH (ref—poor)													1

**p < 0.001, *p < 0.05, *ITA* information technology ability, *ISE* internet self-efficacy, *ICT* information communications technology, *USD* United States dollar, *SRH* self-reported health

Table 3 shows regression coefficients from the consolidated data. After adjusting for the covariates, ITA had a positive association with successful ageing (*β* = 0.29; *t* = 4.22; *p* < 0.001), and its three domains, namely illness avoidance (*β* = 0.20; *t* = 2.66; *p* < 0.001), functioning (*β* = 0.20; *t* = 2.60; *p* < 0.001), as well as engagement with life (*β* = 0.30; *t* = 4.04; *p* < 0.001). This result suggests that higher ITA was associated with higher successful ageing, including functioning, engagement with life, and illness avoidance. ISE was positively associated with successful ageing, illness avoidance, and life engagement but not functioning. The interaction between ISE and ITA had a positive association with successful ageing and its three domains. Compared to the coefficient of ITA (*β* = 0.29) on successful ageing, the coefficient of the interaction term (*β* = 0.50) is 42% larger. The interaction term also had a positive association with illness avoidance (*β* = 0.41), functioning (*β* = 0.31), and engagement with life (*β* = 0.41).

**Table 3:** The associations of information technology ability, internet self-efficacy, and successful ageing (*n* = 1186; Kenya = 350; Nigeria = 260; Ghana = 576)

Variable	Successful ageing	Illness avoidance	Functioning	Engagement with life
	*β*(*t*)	*β(t)*	*β(t)*	*β(t)*
Baseline (non-adjusted) models				
(Constant)	(9.91)**	(5.05)**	(9.94)**	(7.98)**
Internet self-efficacy	0.37(4.12)**	0.45(4.68)**	−0.003(−0.03)	0.42(4.39)**
Information Technology Ability	0.31(4.44)**	0.19(2.56)*	0.23(2.94)*	0.32(4.31)**
Ultimate (adjusted) models with interaction term				
(Constant)	(7.47)**	(5.12)**	(6.41)**	(5.85)**
Internet self-efficacy	0.31(3.40)**	0.41(4.15)**	−0.06(−0.59)	0.36(3.77)**
Information Technology Ability	0.29(4.22)**	0.20(2.66)*	0.20(2.60)*	0.30(4.04)**
ITAxISE	0.50(21.07)**	0.41(18.35)**	0.31(14.35)**	0.41(17.45)**
Gender (ref—men)	0.08(3.47)**	0.07(2.75)*	0.08(3.19)**	0.06(2.40)*
Job tenure (yrs)	0.02(0.88)	0.02(0.63)	−0.001(−0.053)	0.03(1.15)
ICT Training received (ref—no)	−0.07(−3.05)*	−0.06(−2.35)*	−0.056(−2.17)	−0.06(−2.52)*
Income (USD)	0.04(1.38)	0.02(0.64)	0.029(1.04)	0.04(1.34)
Education (yrs)	−0.09(−3.63)**	−0.10(−3.93)**	−0.048(−1.76)	−0.07(−2.87)*
Age (yrs)	−0.03(−1.39)	−0.04(−1.78)	−0.007(−0.27)	−0.03(−1.19)
SRH (ref—poor)	0.10(4.39)**	0.02(0.93)	0.11(4.26)**	0.11(4.27)**

**p < 0.001, *p < 0.05, *ITA* information technology ability, *ISE* internet self−efficacy, *ICT* information communications technology, *USD* United States dollar, *SRH* self−reported health, all statistics in the table are rounded

In Table 4, the associations are disaggregated to show country specific effect sizes. The table indicates that ITA had a positive association with successful ageing and its three domains (*p* < 0.001) in Ghana and Kenya but not in Nigeria, which means that higher ITA was associated with higher successful ageing in only Ghana and Kenya. In the three samples, ITA was more positively associated with successful ageing and its domains at higher ISE.

**Table 4:** A comparison of standardized regression coefficients among Kenya, Nigeria, and Ghana (*n* = 1186; Kenya = 350; Nigeria = 260; Ghana = 576)

Predictors	Kenya				Nigeria				Ghana			
	Successful ageing	Illness avoidance	Functioning	Engagement with life	Successful ageing	Illness avoidance	Functioning	Engagement with life	Successful ageing	Illness avoidance	Functioning	Engagement with life
	*β(t)*	*β(t)*	*β(t)*	*β(t)*	*β(t)*	*β(t)*	*β(t)*	*β(t)*	*β(t)*	*β(t)*	*β(t)*	*β(t)*
Non-adjusted models												
(Constant)	(14.98)**	6.76)**	(11.11)**	(12.05)**	(16.04)**	(10.07)**	(13.04)**	(14.30)**	(14.40)**	(8.11)**	(13.02)**	(14.35)**
ISE	0.45(7.36)**	0.44(6.37)**	0.14(1.93)	0.46(6.43)**	0.22(2.40)*	0.21(2.28)*	0.20(2.15)*	0.14(1.56)	0.32(7.04)**	0.38(7.97)**	0.17(3.42)**	0.30(6.67)**
ITA	0.31(5.09**	0.21(3.22)**	0.46(6.28)**	0.18(2.61)*	0.07(0.79)	−0.06(−0.59)	0.08(0.89)	0.11(1.20)	0.34(7.48)**	0.21(4.51)**	0.30(5.99)**	0.35(7.81)**
Adjusted models with interaction term												
(Constant)	(4.46)**	(0.05)	(3.40)**	(4.33)**	(5.13)**	(4.60)**	(3.47)**	(4.20)**	(7.74)**	(5.55)**	(5.66)**	(7.80)**
ISE	0.43(7.14)**	0.45(6.40)**	0.10(1.29)	0.45(6.14)**	0.25(2.59)*	0.21(2.13)*	0.24(2.47)*	0.17(1.81)	0.41(9.06)**	0.43(8.79)**	0.26(4.96)**	0.40(8.92)**
ITA	0.41(6.87)**	0.37(5.07)**	0.57(7.40)**	0.25(3.25)**	0.07(0.78)	−0.04(−0.38)	0.08(0.83)	0.10(1.08)	0.34(7.79)**	0.22(4.71)**	0.31(6.16)**	0.35(8.08)**
ITAxISE	0.60(13.87)**	0.52(11.04)**	0.43(8.13)**	0.54(10.59)**	0.31(4.74)**	0.21(3.22)**	0.32(4.96)**	0.23(3.45)**	0.58(15.52)**	0.52(13.45)**	0.43(10.40)**	0.57(15.38)**
Gender (ref—men)	0.16(4.36)**	0.10(2.32)*	0.19(4.30)**	0.11(2.58)*	0.11(1.65)	0.14(2.13)*	0.09(1.39)	0.05(0.82)	−0.01(−1.04)	0.004(0.10)	−0.01(−0.38)	−0.01(−0.39)
Job tenure (yrs)	0.08(2.01)*	0.18(3.58)**	0.02(0.44)	0.04(0.82)	0.08(1.08)	0.08(1.10)	0.06(0.88)	0.05(0.73)	−0.04(−1.04)	−0.01(−0.31)	−0.11(−2.75)*	0.01(0.14)
ICT Training received (ref—no)	−0.16(−4.18)**	−0.14(−2.96)*	−0.15(−3.11)*	−0.13(−2.67)*	−0.06(−1.05)	0.03(0.53)	−0.08(−1.30)	−0.09(−1.37)	0.10(2.82)*	0.06(1.59)	0.09(2.11)*	0.11(3.08)*
Income (USD)	0.02(0.51)	−0.01(−0.16)	0.09(1.90)	−0.01(−0.26)	0.07(0.90)	0.10(1.27)	−0.03(−0.43)	0.08(1.07)	0.16(4.83)**	0.12(3.38)**	0.11(2.69)*	0.19(5.53)**
Education (yrs)	−0.09(−2.52)*	−0.04(−0.94)	−0.09(−2.05)*	−0.09(−1.93)	0.03(0.39)	−0.03(−0.37)	0.06(0.84)	0.03(0.39)	−0.221(−6.19)**	−0.19(−4.87)**	−0.14(−3.34)**	−0.24(−6.83)**
Age (yrs)	−0.05(−1.36)	0.05(1.21)	−0.06(−1.28)	−0.08(−1.79)	−0.018(−0.25)	−0.17(−2.28)*	0.04(0.61)	0.04(0.54)	−0.07(−2.14)*	−0.07(−2.09)*	−0.01(−0.24)	−0.09(−2.78)*
SRH (ref—poor)	0.02(0.52)	−0.02(−0.52)	0.02(0.51)	0.03(0.70)	–	–	–	–	0.05(1.35)	−0.03(−0.91)	0.06(1.58)	0.07(2.11)*

**p < 0.001, *p < 0.05, *ITA* information technology ability, *ISE* internet self−efficacy, *SRH* self−reported health, *ICT* information communication technology, *USD* United States dollars, – SRH was removed from the Nigerian models because it was a constant (i.e., all participants reported good health)

**Fig. 3:**
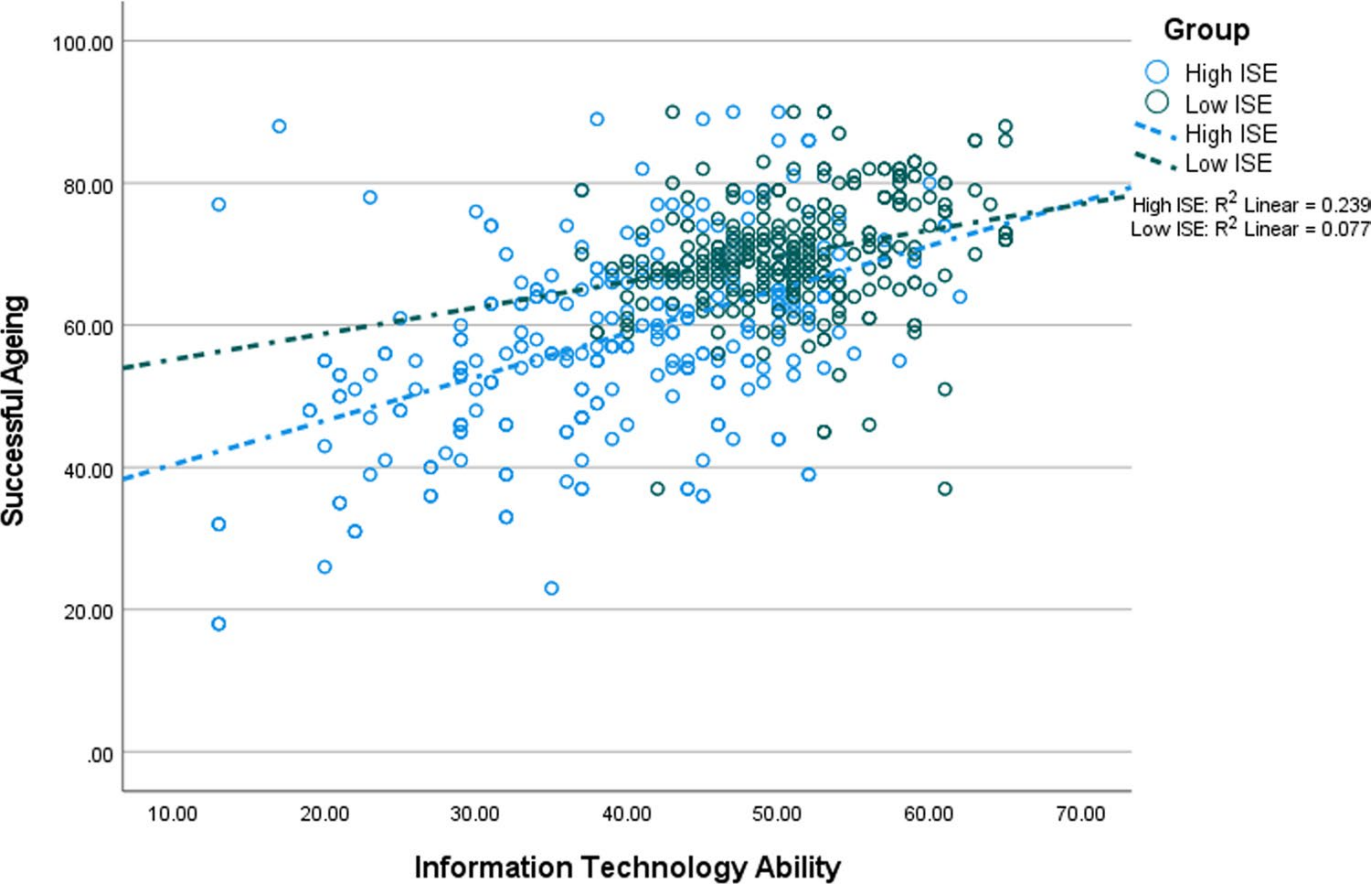
The interaction between information technology and internet self-efficacy on successful ageing (*n* = 1182; low = 591, high = 591)

**Fig. 4:**
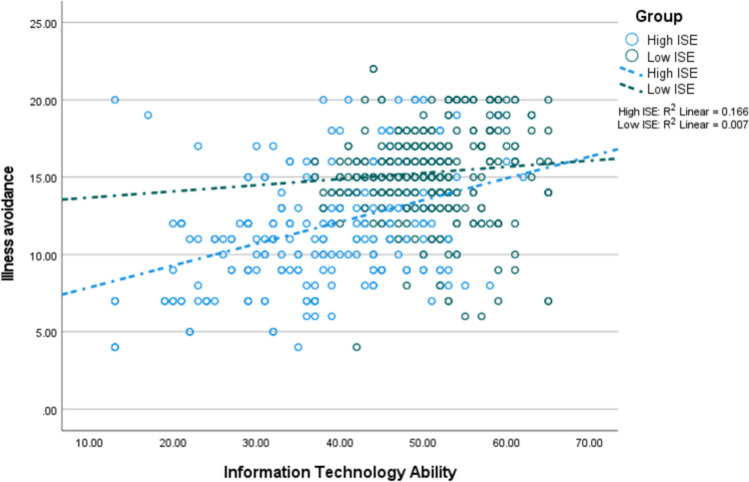
The interaction between information technology and internet self-efficacy on illness avoidance (*n* = 1182; low = 591, high = 591)

**Fig. 5:**
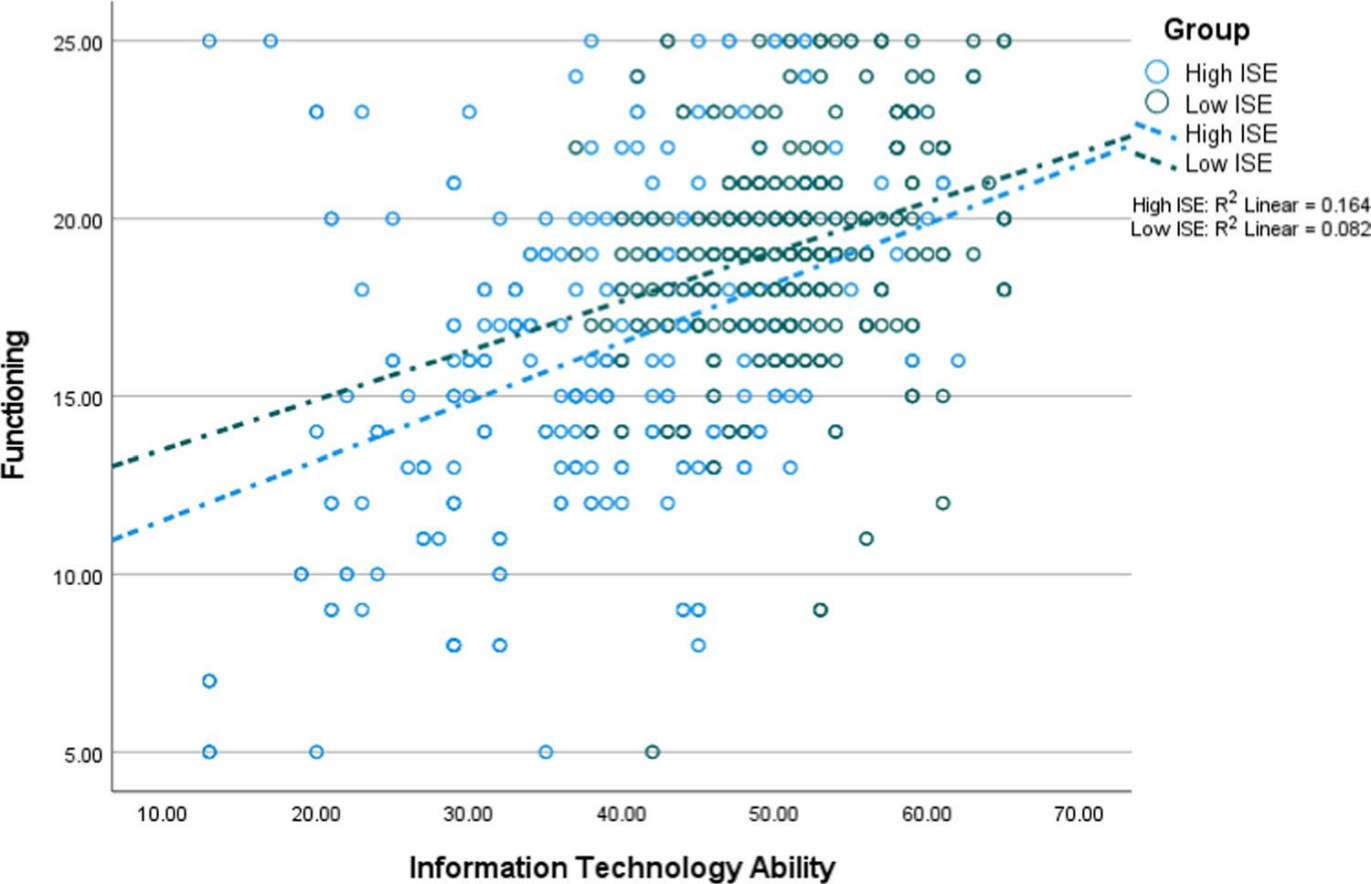
The interaction between information technology and internet self-efficacy on functioning (*n* = 1182; low = 591, high = 591)

**Fig. 6:**
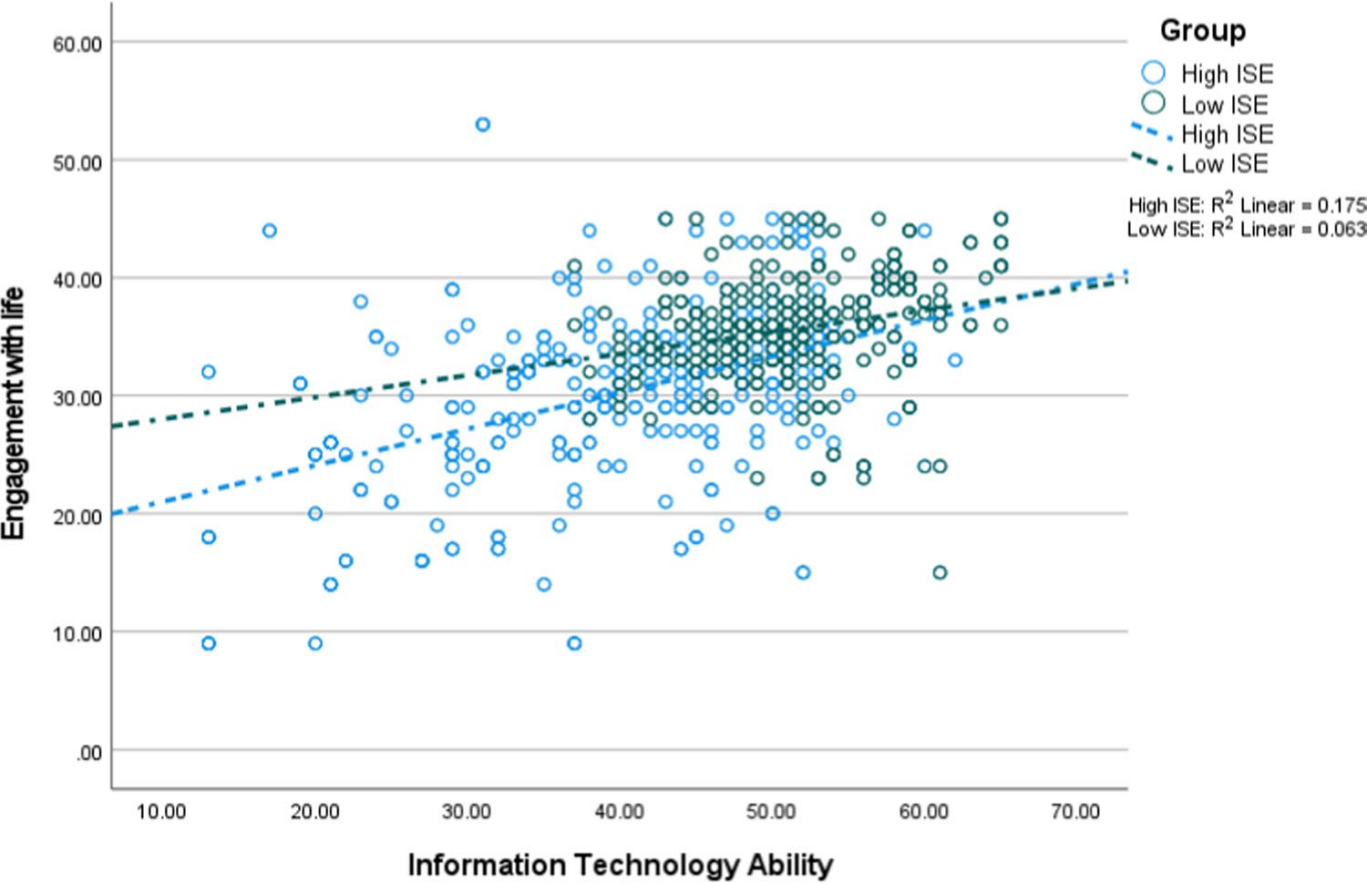
The interaction between information technology and internet self-efficacy on engagement with life (*n* = 1182; low = 591, high = 591)

In Tables 3 and 4, coefficients in the non-adjusted and adjusted models are different due to the presence of the confounders in the adjusted models. Thus, this study would have reported wrong coefficients if it had not adjusted for the confounders. Figures 3, 4, 5 and 6 depict the interaction between ITA and ISE on successful ageing as well as its three domains. Each figure shows that ITA had a positive association with successful ageing, and this relationship was stronger among older adults reporting higher ISE.

## Discussion

This study assessed the association of ITA and its domains on successful ageing with data from three African countries. The potential moderating role of ISE in the foregoing relationship was also examined. Data were collected from participants in three African countries (i.e., Ghana, Kenya, and Nigeria) with a self-reported questionnaire. HLR was utilised to analyse the data.

This study found a positive association of ITA on successful ageing with the consolidated data, which suggests that larger scores of the ability to use information technologies were associated with higher successful ageing. More so, the ability to use information technologies positively influenced illness avoidance and the maintenance of functioning and engagement with life. These confirmed associations were moderate or strong, which emphasises the significance of ITA in successful ageing. The positive association of ITA with engagement with life is supported by a study (Sghaier et al. 2022) that found a positive relationship between ITA and social engagement among older adults including workers in Ghana. Since ITA is necessary for information technology use, our evidence is analogous to results from studies (Aggarwal et al. 2020; Nakagomi et al. 2022) that have tested the association between the use of information technologies (i.e., the internet) and indicators of health, given that all domains of the successful ageing measure used are indicators of health and that our scale measures ITA based on the actual use of information technologies. In Japan, a positive association between internet use and well-being was found (Nakagomi et al. 2022). A systematic review in the UK also reported a positive association between internet use and quality of life in older adults (Aggarwal et al. 2020).

The above result, which supports the first hypothesis (H1) and its sub-hypotheses (H1a, H1b, and H1c), is consistent with the LTC’s view that internal or personal resources such as ITA can enable the individual to exercise primary control over the lifespan by performing behaviours that support health. Since ITA can lead to the use of information technologies in ways that could mar the individual’s health, our positive association of ITA on successful ageing suggest that older adults likely used their ITA to performance tasks that support health. From the standpoints of the LTC, older adults exercised primary control by utilising their abilities to pursue health-supporting goals while avoiding less important goals or the use of information technologies in harmful ways. Older adults seemingly avoided or minimised potential health risks such as stress, anxiety, and bullying reported in the literature (Lim and Choi 2017; van Zoonen and Rice 2017) in their application of their ITA in using information technologies. Worth noting is the consistency of the associations of ITA on successful ageing between Kenya and Ghana; this aspect of our study signifies that the associations could be confirmed in more than one country. Yet, we recognise that our associations (in terms of their strength and direction) may not be consistent across non-African countries due to potential cultural differences between African and non-African countries. For instance, the effect sizes in Nigeria are not consistent with those from Ghana and Kenya. Possibly, the uniqueness of the sample in Nigeria or differences in the sample sizes may have contributed to this inconsistency.

This study further found that ISE enhanced the association of ITA on successful ageing and its three domains on the consolidated data, suggesting that ITA was positively associated with successful ageing at different levels of ISE. This result supports the second hypothesis (H2) and its three sub-hypotheses (i.e., H2a, H2b, and H2c). This result corroborates our recognition of ISE as a unique skill relevant to individual performance and successful ageing in the context of the JCT. Illness avoidance and engagement with life, which were the most strongly associated domains with the interaction between ITA and ISE in Table 3 (*β* = 0.41), could be the most important indicators of individual performance at work. Similar results can be found in Table 4 where the disaggregated evidence is presented. This observation makes sense since no employee can achieve optimum performance without maintaining engagement with life and avoiding ill health (Quintiliani et al. 2010; Zacher 2015). Our result also suggests that the variety of skills possessed by the individual, as asserted by the JCT, can predict performance individually (in terms of the linkage between ITA and successful ageing) or jointly (in terms of the moderating role confirmed).

Our findings have implications for practice, specifically workplace ageing policy. Firstly, our evidence may complement previous research to guide organizations in their adoption of workplace ageing policies intended to enable ageing employees to use information technologies to support health. Workplace programmes aimed at improving ITA and ISE should target and include all employees (not only older employees) since every worker is subject to ageing. Our evidence consolidates previous findings (Lagana 2008) suggesting the importance of investments in training aimed at improving self-efficacy and possibly ITA. Investment in training can be expected to produce desirable outcomes in the way of employees’ illness avoidance, functioning, engagement with life, and the maintenance of optimal performance through information technology use. Moreover, studies (Gutiérrez-Zornoza et al. 2015; Jinsook Kim et al. 2022) have reported the effectiveness of training and human development interventions in enhancing ITA and self-efficacy. As suggested in the literature (Pruchno 2019; Zacher 2015), nevertheless, the aforesaid programmes can only be sustained by incorporating them into the organization’s human resources management policy or adopting a special policy of workplace healthy ageing that prioritises investment in age-friendly workplace interventions. Organizations are likely to lose sight of these programmes if they are not tied to policy and incorporated into routine monitoring.

### Strengths and limitations

This study has some limitations. Firstly, we used non-probability sampling to select the participants since there was no sampling frame for the study. Similarly, the sample was relatively small, especially for each country, and focused on working older adults. Samples were unequal across countries, so the statistical significance of the results was more likely in the larger samples. Our sample comprised only employees in the formal sector, so our evidence would not apply to workers in the informal sector. These shortcomings imply that the generalisability of our results at the national level may be limited. Future researchers are encouraged to address this limitation by utilising nationally representative samples selected with probabilistic sampling methods (e.g., simple random, cluster, and multi-stage sampling methods). Comparing the associations among the countries was beyond the scope of this study, so this study does not tell whether there are significant differences among the three countries in terms of the effect sizes. We adopted a cross-sectional design, which means that this study could not establish causation nor longitudinal associations. Our measures of successful ageing and ITA are subjective, which means these measures were vulnerable to response bias. We could not control for all confounding variables in this study, so we encourage future researchers to control for as many confounding variables as possible. Type of industry and type of job are some potential confounding variables not considered in this study. Future studies may consider these. A more detailed measurement of health in future research is also recommended. Despite these limitations, this study has several strengths.

Noteworthy is our utilisation of samples from three African countries and an assessment of the associations with multiple African samples. This attribute of the study enabled us to establish the potential stability of the associations confirmed, giving stakeholders an idea about the likelihood of ITA and ISE being relevant to successful ageing in the individual countries. Furthermore, we attempted to avoid or minimise common methods bias and confounding, which are the main shortcomings of the cross-sectional design (Asiamah et al. 2021; Kock et al. 2021). Finally, this study is compliant with the STROBE, a research-reporting checklist that maximises research quality if followed (Cuschieri 2019; Sghaier et al. 2022). The way this checklist was applied can be a model for future research. Online appendix C shows sections of this study where relevant items of the STROBE are met.

## Conclusion

Higher ITA can be associated with the avoidance of ill health and the maintenance of functioning and engagement with life among older employees. The positive association of ITA with successful ageing is stronger at higher ISE in the whole sample and in individual samples. A need for organizations and employers to recognise successful ageing as a performance indicator and adopt workplace ageing policies that encourage age-friendly workplace interventions is implied by this study.

## Funding open access

Funding was enabled by the University of Essex.

## Supplementary Information

Below is the link to the electronic supplementary material.

Supplementary file1 (DOC 145 KB)

Supplementary file2 (DOC 58 KB)

Supplementary file3 (DOC 123 KB)

## Data Availability

The data used in this study are available as “GeroTech-Nigeria”, “GeroTech-Kenya”, and “GeroTech-Ghana”.
