# Supplementary material for: Internet self-efficacy moderates the association of information technology ability with successful ageing among older employees in three African samples
Source: Eur J Ageing. 2024 Oct 18;21(1):31. doi: 10.1007/s10433-024-00827-9 (PMC11489396; doi:10.1007/s10433-024-00827-9)
Supplement: Supplementary file 1 — Supplementary file1 (DOC 145 KB) [file 10433_2024_827_MOESM1_ESM.doc]

**Appendix A**

**Appendix A1. The Successful ageing Index or Scale**

On a scale of 1 to 5, where 1 – strongly disagree, 2 – disagree, 3 – somewhat agree, 4 – agree, and 5 – strongly agree, to what extent do you agree or disagree that you experienced the following conditions **OVER THE PAST WEEK**?

| No | Item | 1 | 2 | 3 | 4 | 5 |
| --- | --- | --- | --- | --- | --- | --- |
| Illness avoidance | | | | | | |
| 1 | Bodily pain did not affect my performance of work or other essential tasks. |  |  |  |  |  |
| 2 | I did not use medication or therapy. |  |  |  |  |  |
| 3 | I was healthy enough to move around freely. |  |  |  |  |  |
| 4 | I had good health overall. |  |  |  |  |  |
| Functioning | | | | | | |
| 5 | I had enough energy for daily life. |  |  |  |  |  |
| 6 | I have been sleeping well. |  |  |  |  |  |
| 7 | When I tried to recall familiar names or words, it was not difficult for me to do so. |  |  |  |  |  |
| 8 | I could perform two or more tasks simultaneously, for example, watch TV while discussing something else with another person. |  |  |  |  |  |
| 9 | My body and mind were strong enough to enable me to live independently without having others to take care of me. |  |  |  |  |  |
|  | Engagement with life (Caring engagement) |  |  |  |  |  |
| 10 | I provided concern and support to enrich the lives of nuclear family members (e.g., husband or wife). |  |  |  |  |  |
| 11 | I provided concern and support to enrich the lives of family extended family members (e.g., niece or uncle). |  |  |  |  |  |
| 12 | I provided concern and support to enrich the lives of my neighbours. |  |  |  |  |  |
| 13 | I provided concern and support to enrich the lives of friends, colleagues, or workmates. |  |  |  |  |  |
| 14 | Overall, I was concerned about and supportive to people around me to enrich their lives. |  |  |  |  |  |
| Engagement with life (Productive engagement) | | | | | | |
| 15 | I make financial or productive contribution to my family. |  |  |  |  |  |
| 16 | I make financial or productive contribution to my career and work. |  |  |  |  |  |
| 17 | I make financial or productive contribution to my community or non-profit making organizations. |  |  |  |  |  |
| 18 | Overall, I contributed to society as a whole (including contributions made to your family, your career and work, and the community). |  |  |  |  |  |

Descriptive anchors: 1 – strongly disagree, 2 – disagree, 3 – somewhat agree, and 4 – agree, and 5 – strongly agree.

**Appendix A2: Items for measuring information technology ability**

On a scale of 1 to 5, where **1 – strongly disagree, 2 – disagree, 3 – somewhat agree, 4 – agree,** and **5 – strongly agree**, indicate the extent to which you agree that the following statements apply to you.

| No. | Statement | 1 | 2 | 3 | 4 | 5 |
| --- | --- | --- | --- | --- | --- | --- |
| 1 | In general, I routinely use the Internet to obtain good information. |  |  |  |  |  |
| 2 | I commonly use the Internet to quickly retrieve useful information. |  |  |  |  |  |
| 3 | I would use the Internet to quickly mail attached files to friends. |  |  |  |  |  |
| 4 | I use the Internet because it is easy to get information that relates to my needs. |  |  |  |  |  |
| 5 | I would use the Internet because getting required information is inexpensive. |  |  |  |  |  |
| 6 | I could share ideas and thoughts on specific topics through package software. |  |  |  |  |  |
| 7 | I could apply word-processing software in document editing. |  |  |  |  |  |
| 8 | I could use package software to publish documentation on the Internet. |  |  |  |  |  |
| 9 | I could describe the package software functions. |  |  |  |  |  |
| 10 | I like to experiment with new technologies. |  |  |  |  |  |
| 11 | In general, I am hesitant to try out new technologies. |  |  |  |  |  |
| 12 | If I heard about new technologies, I would look for ways to experiment with them. |  |  |  |  |  |
| 13 | Among my peers, I am usually the first to try out new technologies. |  |  |  |  |  |

Descriptive anchors: 1 – strongly disagree, 2 – disagree, 3 – somewhat agree, 4 – agree, and 5 – strongly agree; Domains: items 1-5 measure internet use assessment; items 6-9 measure packaged software use assessment, and items 10-13 measure personal innovativeness attitude.

**Appendix A3. Items for measuring internet self-efficacy**

On a scale of 1 to 6 (**where 1 – strongly disagree; 2 – moderately disagree; 3 – slightly disagree; 4 – slightly agree, 5 – moderately agree, and 6 – strongly agree**), indicate the extend of:

| SN |  | 1 | 2 | 3 | 4 | 5 | 6 |
| --- | --- | --- | --- | --- | --- | --- | --- |
| 1 | understanding terms/words relating to Internet hardware. |  |  |  |  |  |  |
| 2 | understanding terms/words relating to Internet software. |  |  |  |  |  |  |
| 3 | describing functions of Internet hardware |  |  |  |  |  |  |
| 4 | trouble shooting Internet problems. |  |  |  |  |  |  |
| 5 | explaining why a task will not run on the Internet. |  |  |  |  |  |  |
| 6 | using the Internet to gather data. |  |  |  |  |  |  |
| 7 | learning advanced skills within a specific Internet problem. |  |  |  |  |  |  |
| 8 | turning to an on-online discussion group when help is needed. |  |  |  |  |  |  |

Descriptive anchors: 1 – strongly disagree; 2 – moderately disagree; 3 – slightly disagree; 4 – slightly agree, 5 – moderately agree, and 6 – strongly agree
