# Supplementary material for: Internet self-efficacy moderates the association of information technology ability with successful ageing among older employees in three African samples
Source: Eur J Ageing. 2024 Oct 18;21(1):31. doi: 10.1007/s10433-024-00827-9 (PMC11489396; doi:10.1007/s10433-024-00827-9)
Supplement: Supplementary file 2 — Supplementary file2 (DOC 58 KB) [file 10433_2024_827_MOESM2_ESM.doc]

Appendix B. Assumptions governing the use of hierarchical linear regression and steps followed to assess and meet them

| # | Assumption | Step | Result | Decision |
| --- | --- | --- | --- | --- |
| 1 | Normality of the data associated with the dependent variable | Assessed normality with the Shapiro-Wilk’s test of normality | The Shapiro-Wilk’s test produced a non-significant result at p >0.05 (Asiamah et al., 2022). | Normality was established |
| 2 | Linearity of the associations | We plotted standardized residuals against standardized predicted values of the dependent variable in HLR analysis models through which the hypotheses were tested. | The graph shows a linear cluster of values and a straight line as recommended (Asiamah et al., 2022) | Assumption or condition was met |
| 3 | Independence of regression errors | Durbin Watson statistics were generated for all the HLR models fitted. | Durbin-Watson statistic was approximately 2 as recommended (Asiamah et al., 2022) | The assumption was met |
| 4 | Multi-collinearity | Tolerance values were computed through the HLR models through which the hypotheses were assessed | The tolerance values are >0.2 as recommended (Asiamah et al., 2022) | The assumption was met |
| 5 | Homogeneity of variances | We plotted standardized residuals against standardized predicted values of the dependent variable in the HLR models through which the hypotheses were tested | The graphs produced a satisfactory pattern as recommended (Asiamah et al., 2022) | The assumption was met |

**Note**: HLR – hierarchical linear regression
